# Management of Primary Synovial Osteochondromatosis in the Ankle Joint With a Combined Posterior-Anterior Arthroscopic Procedure: A Case Report

**DOI:** 10.7759/cureus.60843

**Published:** 2024-05-22

**Authors:** Rui Correia Cardoso, Filipe Malheiro, Bruno Pereira

**Affiliations:** 1 Orthopaedics and Traumatology Surgery, Centro Hospitalar Baixo Vouga, Aveiro, PRT; 2 Foot and Ankle, Clinica Espregueira Mendes, Porto, PRT

**Keywords:** synovectomy, arthroscopy, loose bodies, synovial osteochondromatosis, ankle

## Abstract

Primary synovial osteochondromatosis (PSO), a seldom-seen synovial proliferative disease involving chondral metaplasia, presents a unique challenge when affecting the ankle joint. Controversy exists regarding whether a combined posterior-anterior approach with total synovectomy is necessary to avert recurrence or malignancy.

An 18-year-old Caucasian male presented to the outpatient clinic with clinical and imaging findings indicative of a stage III PSO. The surgical intervention involved a combined posterior-anterior arthroscopic approach with the removal of multiple loose bodies and complete synovectomy, resulting in complete relief of symptoms without recurrence at the 12-month follow-up. Pathological examination confirmed the diagnosis.

The management of PSO in the ankle joint using a combined posterior-anterior arthroscopic approach with complete synovectomy demonstrated effectiveness in this case. Regular follow-ups are essential for monitoring long-term outcomes and detecting potential recurrence or malignant transformation.

## Introduction

Primary synovial osteochondromatosis (PSO) is an uncommon, slowly progressive, and self-limited condition with an etiology yet to be clarified. The pathological process involves synovial cell cartilaginous metaplasia, leading to the formation of multiple cartilaginous nodules [1-3]. Due to synovial fluid nourishment, the cartilaginous bodies may ossify, proliferate, and become loose bodies within the joint [2,4]. Typically monoarticular, PSO most frequently affects large joints, with the ankle rarely being involved [5]. Without intervention, PSO may result in degenerative alterations within the impacted joint [5], with the potential for progressing into chondrosarcoma [4,6]. Treatment decisions primarily depend on the patient’s age, symptoms, and disease stage, with surgery often emerging as the preferred choice. Both open arthrotomy and arthroscopic techniques have been employed [1,7-9]. However, controversy exists regarding whether a combined posterior-anterior approach, along with total synovectomy, is necessary to prevent recurrence or malignancy [3,9,10].

In this case report, we present a young adult with a right ankle PSO in Milgram phase III [11]. This patient underwent treatment with a combined posterior-anterior ankle arthroscopy with complete synovectomy and loose body removal. Additionally, we discuss the potential benefits of this treatment approach.

## Case presentation

An 18-year-old Caucasian male patient presented to the outpatient clinic for the evaluation of progressive mechanical pain, swelling and decreased range of motion in his right ankle for more than one year. The patient had a history of an ankle sprain after inverting his right ankle while playing soccer. There was no reported family history of bone or joint disorders, and the patient had no known comorbidities.

On physical examination, the patient presented with swelling of the ankle and tenderness along the anterior ankle joint line. Palpable loose bodies were palpated within the capsule of the anterior right ankle. Range of motion throughout the right ankle joint was limited, accompanied by crepitus. Anterior positive impingement was observed, and no instability was verified. Dermatological, vascular, and neurological assessments of the right foot, ankle and lower extremity showed no abnormalities. 

**Figure 1:**
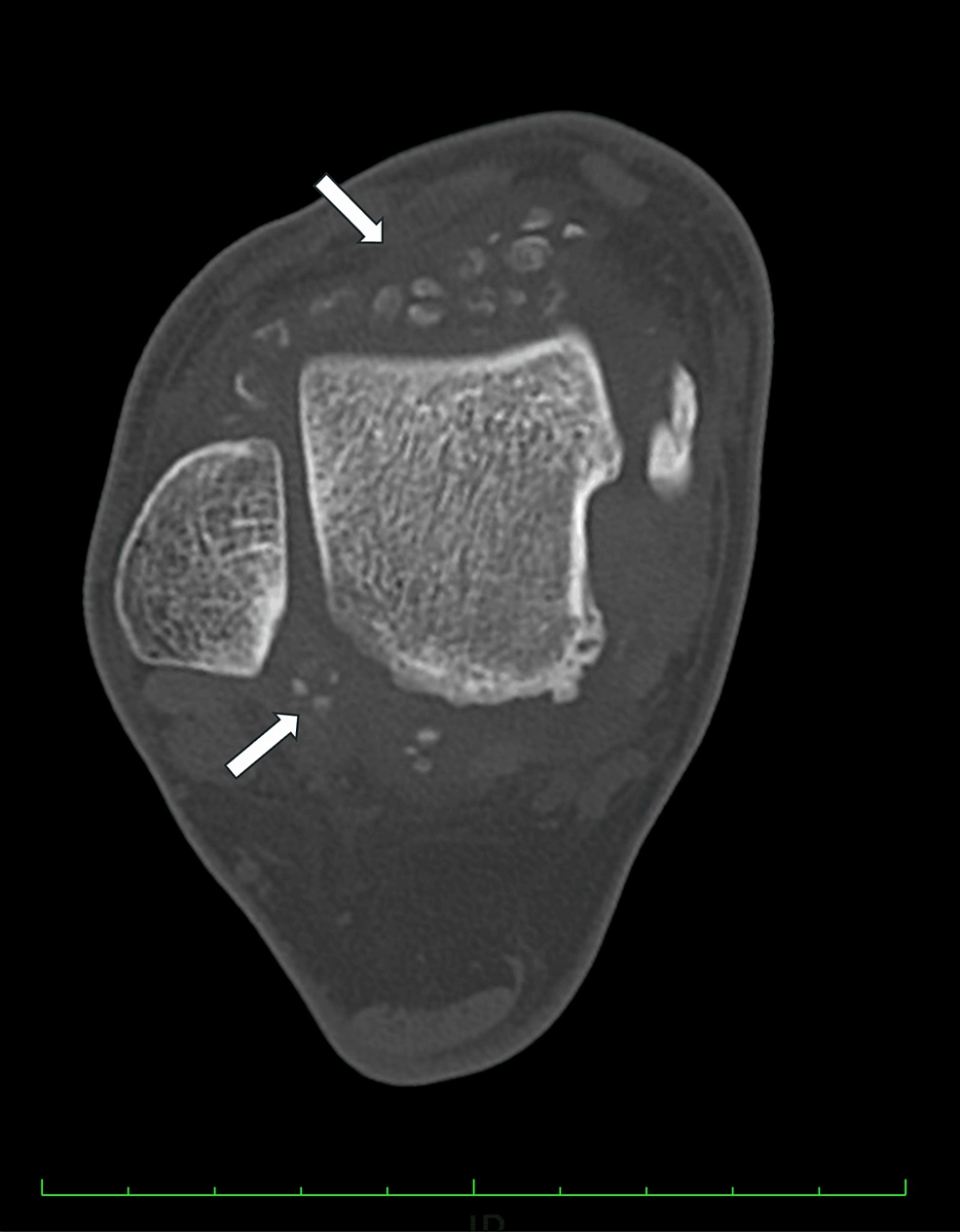
Preoperative axial CT of the right ankle.

**Figure 2:**
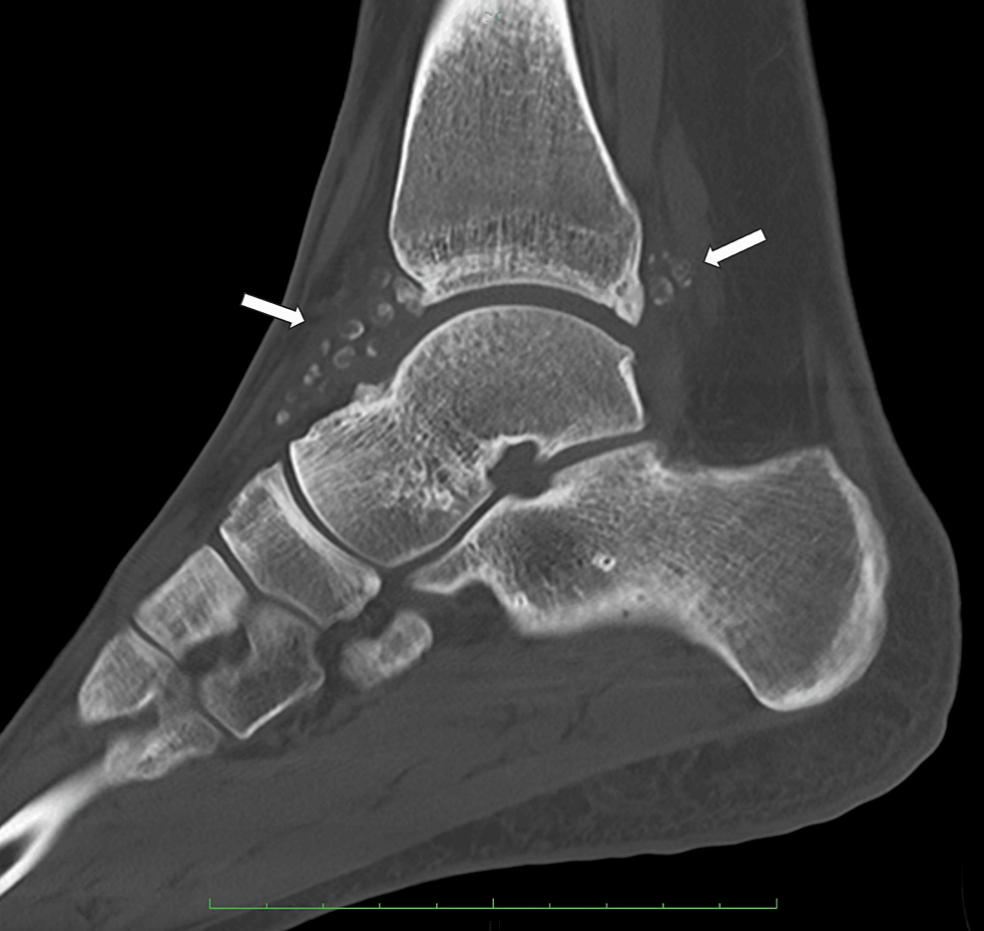
Preoperative sagittal CT of the right ankle.

**Figure 3:**
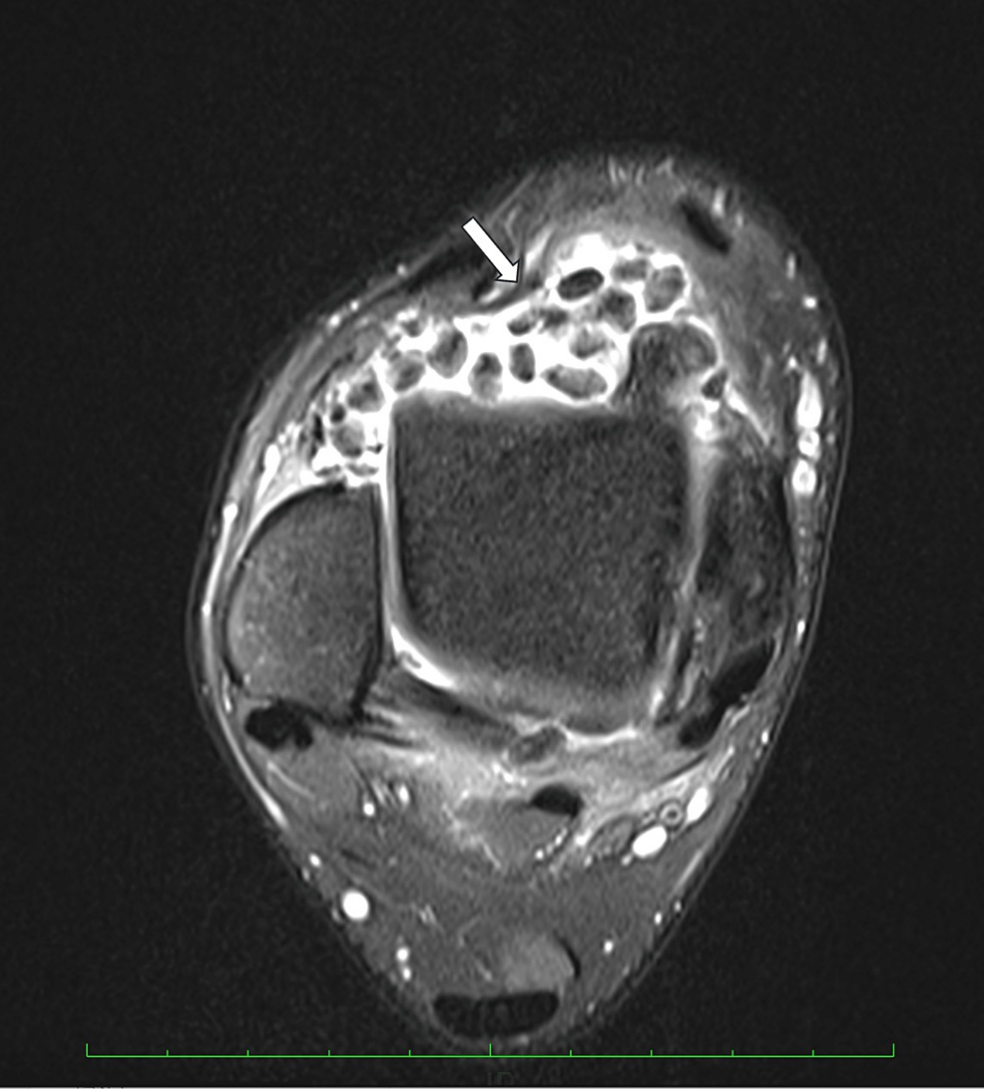
Preoperative axial dual phase MRI of the right ankle.

**Figure 4:**
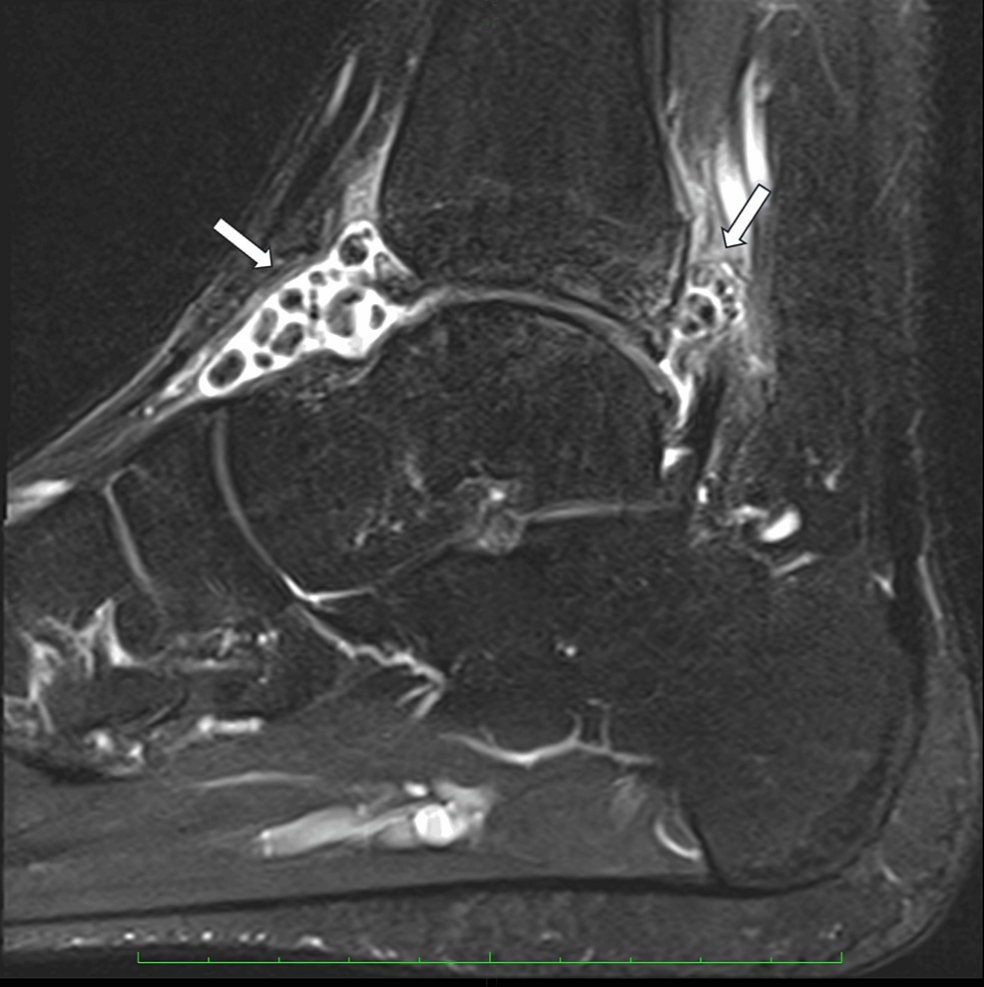
Preoperative sagittal T2 MRI of the right ankle.

Plain radiography showed multiple nodules in both the anterior and posterior compartments of the ankle joint. Computed tomography and magnetic resonance imaging revealed several ossified loose bodies with well-defined borders, measuring up to 8.4mm in diameter, accompanied by synovitis in the ankle joint (Figures 1-4), consistent with PSO in Milgram phase III [11].

Operative technique

**Figure 5:**
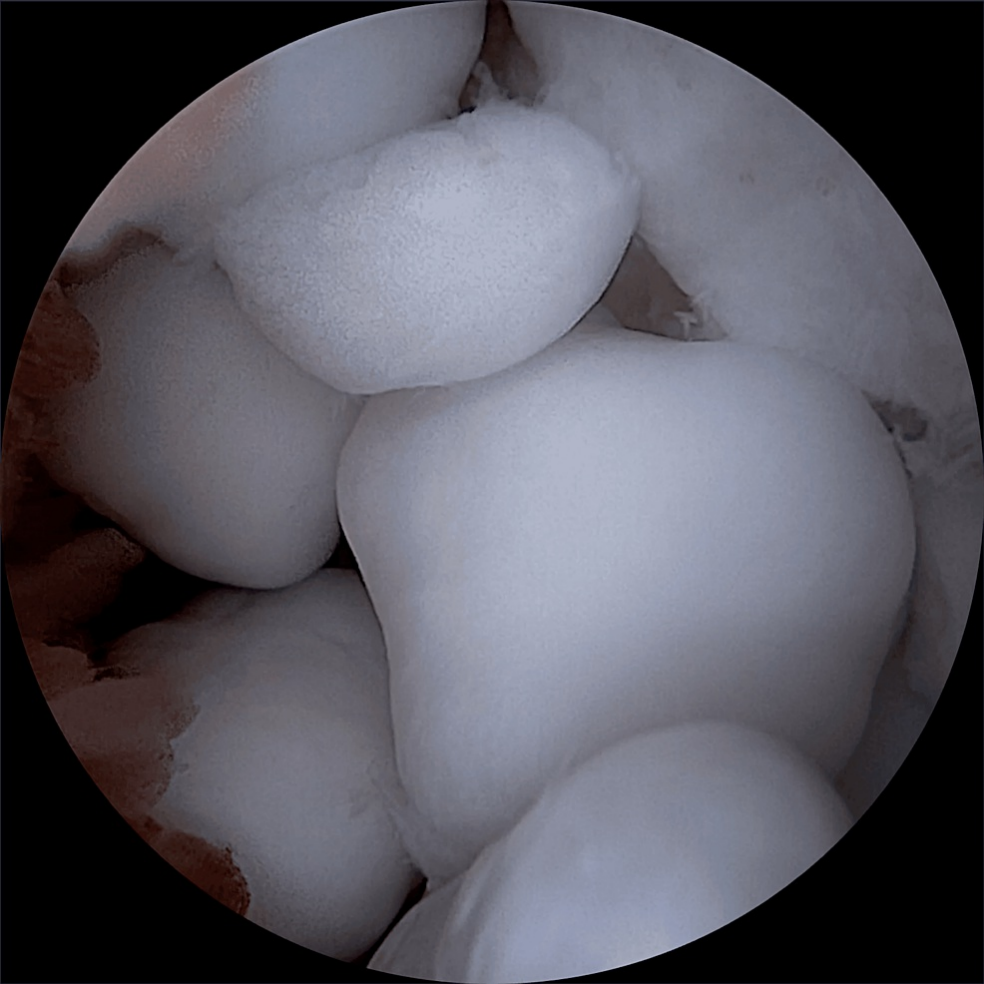
Arthroscopic view of the anterior ankle compartment showing multiple loose bodies.

**Figure 6:**
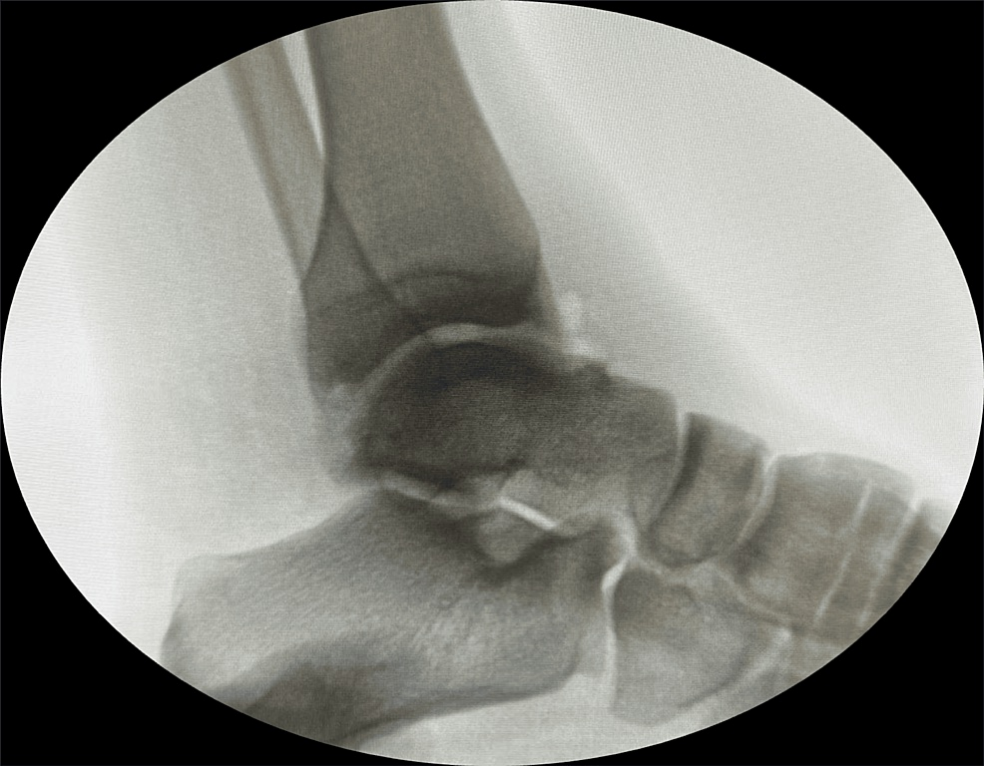
Intraoperative fluoroscopy confirms the complete removal of radio-opaque loose bodies.

**Figure 7:**
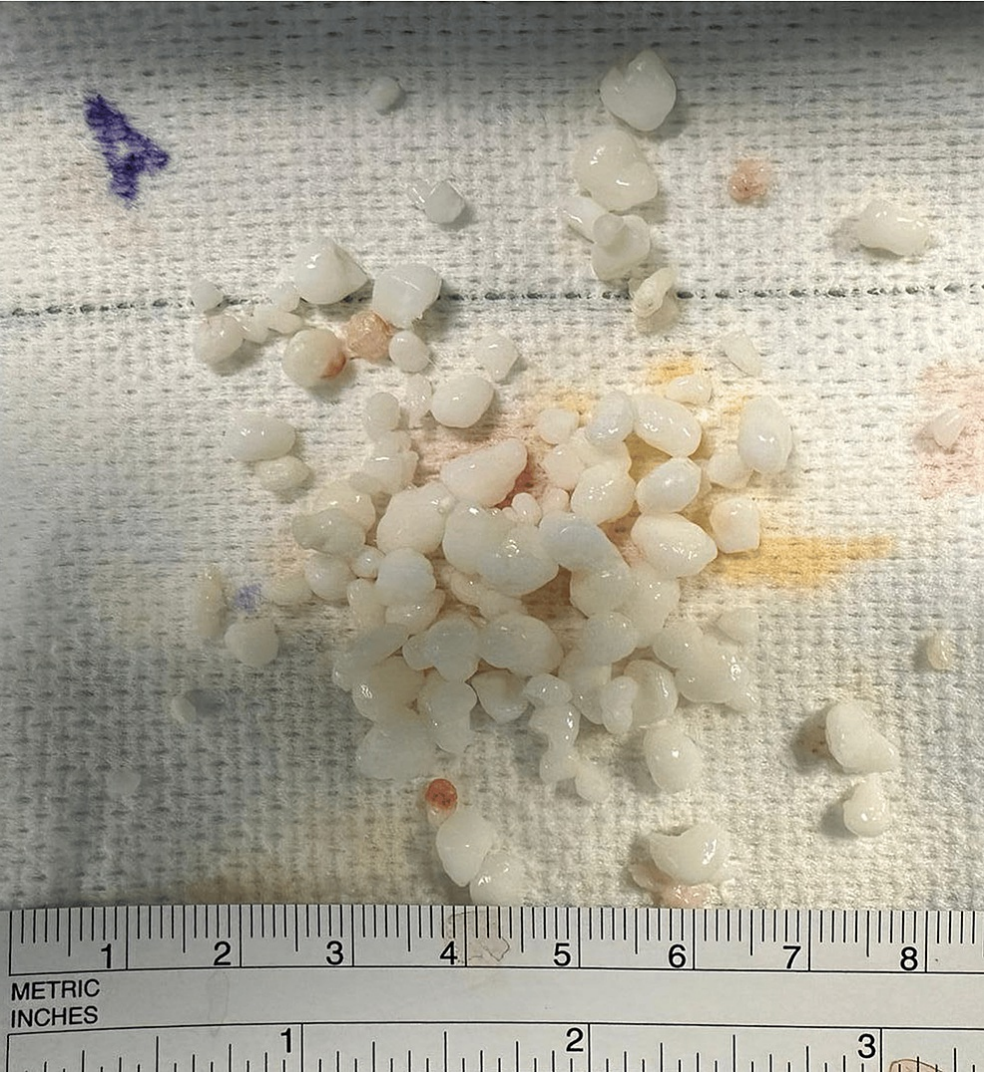
Intraoperative image showing multiple loose bodies removed arthroscopically from the ankle joint.

The surgery was performed under peripheral nerve block and general anesthesia, with the use of a tourniquet. The patient was initially placed in the prone position for a posterior ankle arthroscopy through posteromedial and posterolateral portals, following the technique described by van Dijk [12]. Subsequently, the patient was repositioned in the supine position for an anterior ankle arthroscopy through standard anteromedial and anterolateral portals. An arthroscope measuring 4.0mm in diameter and angled at 30 degrees, along with and arthroscopic pump, was used. Distraction was not employed. A routine arthroscopic assessment of both the posterior and anterior compartments of the ankle joint revealed numerous loose bodies and a hypertrophic synovial lining. The removal of loose bodies and complete synovectomy of both compartments were carried out (Figure 5). Throughout the procedure, samples of proliferative synovial tissue and loose bodies were obtained from both compartments for histopathologic analysis, and intraoperative fluoroscopy confirmed the removal of all osteochondroid fragments (Figures 6-7). Skin incisions were closed using 3/0 nonabsorbable suture.

Post-operatively

Following surgery, the patient was encouraged to begin range-of-motion exercises with partial weight-bearing, using crutches as tolerated, from the first day. At the two-week follow-up, sutures were removed, and the patient was cleared for full weight-bearing. Subsequent follow-up appointments were scheduled for six weeks and 10 weeks after the surgery, during which the patient did not report any limitations in range of motion or weight bearing. At the patient’s most recent follow-up, 12 months after the surgery, no complaints were noted, and radiographs showed no recurrence of synovial chondromatosis loose bodies. Histopathological examination confirmed that the osteochondroid tissue fragments were consistent with osteochondromatosis.

## Discussion

The treatment goal of PSO focuses on addressing pain, restoring joint function, and preventing early osteoarthritis. Treatment decisions are primarily influenced by the patient’s age, symptoms, and disease stage, with surgery often emerging as the preferred choice in Milgram phases II and III [11].

The preference for arthroscopy over open arthrotomy is justified by advantages such as easier access to the entire articular surface, lower morbidity, faster recovery, and increased patient satisfaction. Conversely, opting for open arthrotomy to remove numerous loose bodies in both anterior and posterior compartments would require extensive simultaneous anterior and posterior approaches, thereby increasing the risk of arthrofibrosis and prolonging the rehabilitation period [8].

The proposed combined posterior-anterior arthroscopic technique with complete synovectomy emerges as an optimal surgical approach [7]. Some authors exclusively endorse the anterior approach for ankle PSO operative treatment. However, the main disadvantage of this approach is the mandatory use of a distraction device and the difficulty associated with removing all loose bodies or inflamed synovium from the posterior compartment. Consequently, recurrences with posteriorly localized loose bodies have been reported following anterior-only approaches [6,13]. A potential limitation of the combined posterior-anterior approach involves the need for position switching during the surgery, potentially prolonging the procedure’s duration and raising the risk of infection. However, higher infection rate has not been observed in previous literature [7,14,15].

Late-stage presentations, as demonstrated in this case, suggest the subsidence of active synovitis, prompting some authors to argue the sufficiency of loose body removal alone at this stage [13,16]. Nevertheless, recent evidence supporting complete synovectomy to prevent recurrence and malignancy introduces a crucial consideration. The reported recurrence rate of PSO after loose body removal alone, ranging from 3% to 60%, and up to 8% after a concomitant synovectomy, underscores the significance of thorough synovial tissue management [3]. This higher recurrence rate with loose body removal alone is thought to be due to active synovium remaining or the presence of the stimulus that caused the metaplasia, with some authors going further, asserting that incomplete synovectomy results in a higher recurrence rate compared to complete synovectomy [10]. Irrigation of the joint during surgery with 3% H2O2 has been proposed as a potential additional measure to prevent recurrence of PSO. This method acts as a chemical cauterization and is not thought to be associated with additional surgical complications [17].

Malignancy transformation of PSO to chondrosarcoma is present in approximately 5% of the cases [4,6,8], and evidence suggests that total synovectomy decreases the risk of malignant transformation by reducing recurrence rates [1,18].

Most recurrences occur after more than five years after surgery [7] and malignant transformation typically takes place many years after operative treatment [4,6,8], therefore we recommend counseling patients in this regard and offering periodic follow-ups to detect any recurrence or malignancy.

## Conclusions

PSO, though uncommon, can lead to progressive mechanical pain and joint dysfunction, emphasizing the importance of prompt diagnosis and appropriate surgical intervention. The management of PSO in the ankle joint, employing a combined posterior-anterior arthroscopic approach with complete synovectomy, demonstrated effectiveness in this case. Regular follow-ups are essential for monitoring long-term outcomes and detecting potential recurrence or malignant transformation.
